# GPBAR1 is associated with asynchronous bone metastasis and poor prognosis of hepatocellular carcinoma

**DOI:** 10.3389/fonc.2022.1113785

**Published:** 2023-01-23

**Authors:** Nan Chen, Jieqing Wang, Lei Zhou, Baiqiang Hu, Yinzhong Chen, Zhuangchen Zhu

**Affiliations:** ^1^ Department of Orthopaedic Surgery, The Second Affiliated Hospital of Shandong First Medical University, Tai'an, Shandong, China; ^2^ Department of Pharmacy, The Affiliated Taian City Central Hospital of Qingdao University, Tai'an, Shandong, China; ^3^ Department of Orthopaedic Surgery, Yantai Yuhuangding Hospital, Yantai, Shandong, China

**Keywords:** GPBAR1, asynchronous bone metastasis, HCC, prognosis, biomarker

## Abstract

**Background:**

Hepatocellular carcinoma (HCC) is the second leading cause of cancer-related death in China. Asynchronous metastasis is the main reason for HCC recurrence, but the current assessment of HCC metastasis and prognosis is far from clinically satisfactory.

**Materials:**

In our study, we investigated the expression of G-protein-coupled bile acid receptor (GPBAR1) in HCC tissues and tumor-adjacent tissues by qRT-PCR and immunohistochemistry. The associations between GPBAR1 expression, clinicopathological factors, and asynchronous metastases were assessed by the Chi-square test. The overall survival curves of different variables were plotted with the Kaplan–Meier method, and the statistical significance between different subgroups was analyzed with the log-rank test. The independent prognostic factors were identified by the Cox regression hazard model.

**Results:**

GPBAR1 was more highly expressed in HCC tissues than in tumor-adjacent tissues. GPBAR1 expression in HCC was significantly higher than that in liver cirrhosis, followed by normal liver tissues. GPBAR1 was significantly associated with poor prognosis in HCC and can be regarded as an independent prognostic biomarker. Interestingly, GPBAR1 expression in HCC was significantly correlated with asynchronous metastasis to the bone but not to the liver or lung.

**Conclusions:**

GPBAR1 was found to be an independent, unfavorable prognostic factor of HCC, as well as an indicator of asynchronous bone metastasis but not liver or lung metastases. Our results could provide a new aspect for HCC metastasis studies and help identify high-risk HCC patients, which helps ameliorate the prognostic assessment of HCC.

## Introduction

Hepatocellular carcinoma (HCC) is the third leading cause of cancer-related death and the second leading cause of cancer death worldwide (1). China has the most severe hepatitis B virus (HBV) prevalence, and the incidence of HCC has doubled in the UK and tripled in the USA over the past three decades (2). More than one million deaths from HCC are predicted for 2030 by the WHO (2, 3). In addition to surgical resection, several new treatment approaches to HCC have been developed, such as trans-arterial chemoembolization, tyrosine kinase inhibitors, and immunotherapy (4). However, the prognosis for HCC is still very dismal. The current prognostic assessment of HCC is far from clinically satisfactory. Several proposals have been made for better predicting outcomes and guiding the treatment (5). New biomarkers and drug targets are still urgently needed to improve patient stratification for precision treatment.

Hepatocytes produce bile acids (BAs) to help the absorption of cholesterol, fat-soluble vitamins, and lipids. In addition, BAs participate in plenty of physiological processes such as glucose homeostasis, lipid and energy metabolism, and immune response (6). Deregulation of BA homeostasis is also reported to be involved in tumorigenesis and tumor progression (7). BAs bind with BA receptors with different affinities and mediate BA-specific signaling (8, 9). BA receptors consist of nuclear receptors and membrane-bound G protein-coupled receptors. The former contains farnesoid X receptors (FXR), pregnane X receptors (PXR), and vitamin D receptors (VDR) (10); the latter is composed of G-protein-coupled bile acid receptor (GPBAR1, also known as TGR5) and sphingosine-1-phosphate receptor 2 (S1PR2) (11).

Among all the BA receptors, GPBAR1 is widely expressed in human tissues (12). High GPBRA1 expression is reported in the gallbladder, placenta, spleen, lung, liver, intestine, kidney, adrenal glands, adipose tissue, etc. (13). GPABR1 plays an essential role in regulating energy homeostasis, bile acid homeostasis, and glucose metabolism (14, 15). Emerging evidence has shown the involvement of GPBAR1 in cancer progression. GPBAR1 is upregulated and promotes the progression of gallbladder cancer, cholangiocarcinoma, and lung cancer (16–18). The expression of GPBAR1 and its clinical significance, especially the prognostic significance in HCC, are still unknown.

In our study, we investigated the expression of GPBAR1 in HCC tissues and tumor-adjacent tissues. Moreover, we evaluated the clinical significance of GPBAR1 in HCC by analyzing its correlation with clinicopathological factors, overall survival (OS) rate, and asynchronous metastases to the liver, lung, and bone.

## Materials and methods

### HCC cohort and ethics

With the patients’ prior consent, 10 pairs of HCC tissues and tumor-adjacent tissues were obtained from surgery. The other cohort contains 201 HCC patients who underwent radical surgery from January 2014 to December 2019 in the Second Affiliated Hospital of Shandong First Medical University and The Affiliated Taian City Central Hospital of Qingdao University. Formalin-fixed and paraffin-embedded specimens were obtained after the approval of patients or patients’ relatives. The OS time was determined by the time interval from the surgery date to the date of the last follow-up or the patient’s death. HCC staging was according to the eighth TNM staging system. Asynchronous metastasis was determined by biopsy or a clinical diagnosis, including elevated AFP and classic imaging manifestations. Patients with asynchronous metastasis received standard treatments according to the HCC treatment guideline (19, 20). For patients with liver metastasis, topical treatments such as tumor ablation or transcatheter arterial chemoembolization (TACE) were performed, supplemented with systemic treatments such as target therapy or immune checkpoint inhibitor (ICI) treatment. Patients with bone and lung metastasis mainly received radiotherapy or systemic treatment.

All experiments were performed with the approval of the Ethics Committees of the Second Affiliated Hospital of Shandong First Medical University and The Affiliated Taian City Central Hospital of Qingdao University.

### Immunohistochemistry and evaluation

GPBAR1 expression in HCC was detected by immunohistochemistry (IHC) with the streptavidin peroxidase complex method. In brief, the specimens were first soaked in boiled EDTA (pH = 9.0) buffer for antigen retrieval and then incubated in 3% hydrogen peroxide to inactivate the endogenous peroxidase. After that, 5% bovine serum albumin was applied to incubate specimens to block unspecific antigen binding. The primary antibody for GPBAR1 (Abcam, ab72608, Cambridge, UK) was administrated to incubate the tissues overnight at 1:100 concentrations, followed by the secondary antibody labeled with biotin (Zsbio, Beijing, China) treatment for 30 min at room temperature. The final antigen vision was performed with 3,3-diaminobenzidine (DAB) solution (Zsbio).

The slides were evaluated by two senior pathologists, and the IHC results were semiquantified by scores of the staining intensity and the positively stained cell percentage. The final IHC score was defined as the number of the score (staining intensity) multiplied by the score (positively stained cell percentage). Scores of staining intensity were classified as 0 (negative staining), 1 (weak staining), 2 (moderate staining), and 3 (strong staining). The scores of positively stained cell percentage were determined as 1 (<25% positive cells), 2 (25%–50% positive cells), 3 (50%–75% positive cells), and 4 (75%–100% positive cells). The final IHC score varied from 0 to 12. The cohort was divided into subsets with different GPBAR1 expressions by the cutoff of the IHC score, which was identified by the receiver operating characteristic (ROC) curve. In our study, the cutoff for the GPBAR1 IHC score in HCC was 2.5.

### Quantitative real-time PCR

The total mRNA of HCC tissues and corresponding tumor-adjacent tissues was extracted by the TRIzol reagent (Thermo Fisher, Waltham, MA, USA). cDNA synthesis was accomplished by a reverse transcriptase kit (TOYOBO, Osaka, Japan), and real-time PCR was performed with SYBR Green Master Mix (Roche, Basel, Switzerland) and Light Cycler Roche 480 PCR instrument. The Ct value of GAPDH was used as an internal control, and the 2^−ΔΔCt^ method was applied to compare the difference between HCC and tumor-adjacent tissues. The primer sequences for GPBAR1 and GAPDH were as follows: GPBAR1 sequence (5′–3′): forward: CCCAGGCTATCTTCCCAGC; reverse: GCCAGGACTGAGAGGAGCA. GAPDH sequence (5′–3′) forward: GCACCGTCAAGGCTGAGAAC; reverse: TGGTGAAGACGCCA GTGGA.

### Statistical analysis

All data were analyzed with SPSS 22.0 software (SPSS, Chicago, IL, USA). The association between GPBAR1 expression, clinicopathological factors, and asynchronous metastases was assessed by the Chi-square test. OS curves were plotted with the Kaplan–Meier method, and the statistical significance between different subgroups was analyzed with the log-rank test. The independent prognostic factors were identified by the Cox regression hazard model. *p*-value <0.05 was considered statistically significant.

## Results

### Expression of GPBAR1 in HCC

**Figure 1:**
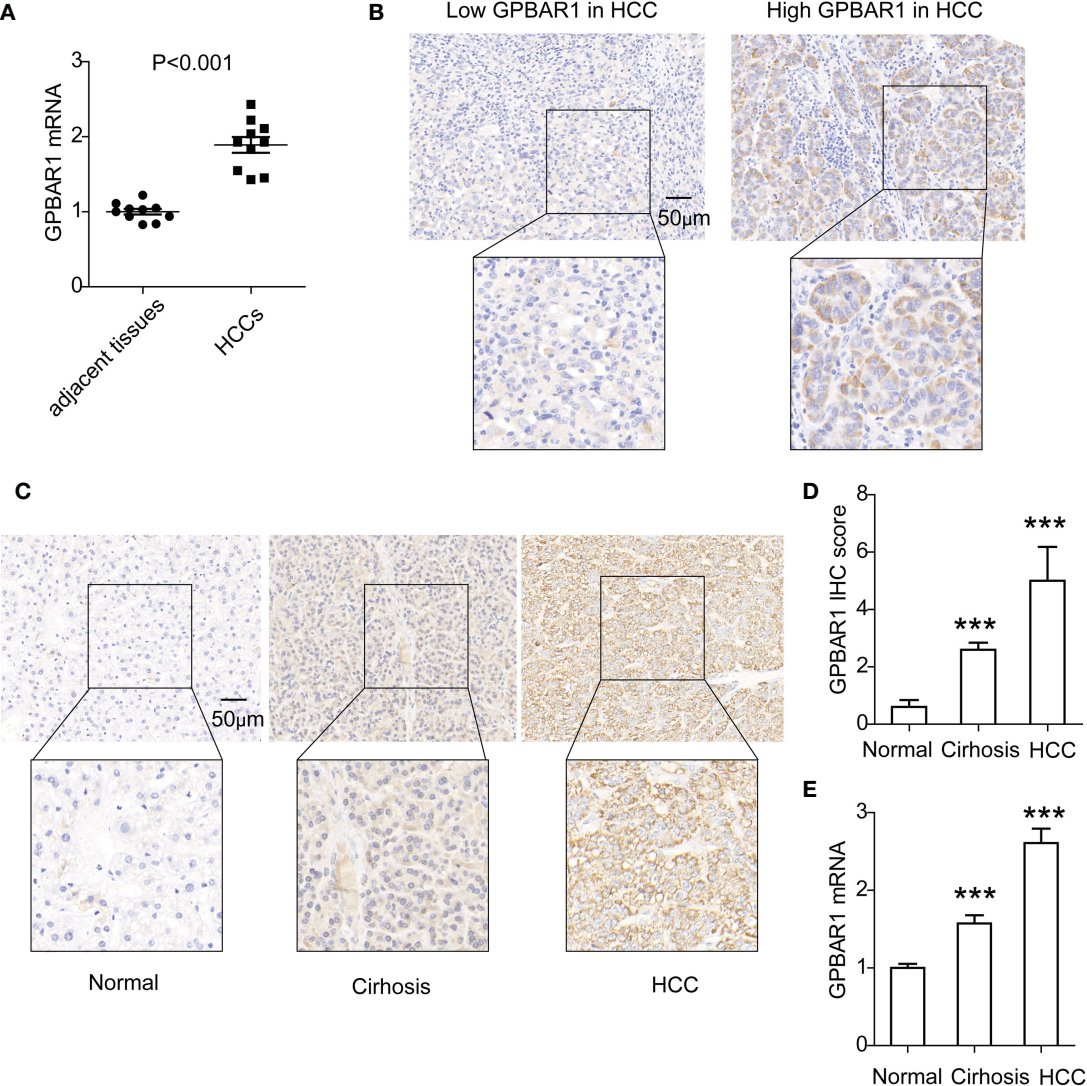
Expression of GPBAR1 in HCC and adjacent tissues. **(A)** The mRNA level of GPBAR1 was assessed by qPCR in 10 pairs of HCC and tumor-adjacent tissues. The statistical significance is analyzed by a paired *t*-test. **(B)** GPBAR1 expression in HCC was detected by IHC, which divided the cohort into GPBAR1^low^ and GPBAR1^high^ subsets. **(C, D)** GPBAR1 expressions in normal liver tissues, liver cirrhosis, and HCC tissues were detected with IHC **(C)**. The IHC scores of GPBAR1 in these tissues were evaluated and compared **(D)**. **(E)** GPBAR1 expressions in normal liver tissues, liver cirrhosis, and HCC tissues were detected with qRT-PCR. The average level of GPBAR1 mRNA in normal liver tissues was set as a baseline for data normalization. ^***^ *p* < 0.001 with a one-way ANOVA. The data were from at least three independent experiments.

The expressions of GPBAR1 in HCC and tumor-adjacent tissues were first assessed by mRNA. A total of 10 pairs of HCC tissues and corresponding tumor-adjacent tissues were detected. In HCC tissues, GPBAR1 expression was significantly higher than that in tumor-adjacent tissues, indicating a potential oncogenic role for GPBAR1 in HCC (**Figure 1A**). Moreover, we investigated the expression of GPBAR1 in the HCC tissues of our cohort, consisting of 201 HCC patients with IHC. GPBAR1 was mainly expressed in the cell membrane and cytosol of HCC (**Figure 1B**). GPBAR1 expression was evaluated by IHC score, which divided the cohort into GPBAR1^low^ and GPBAR1^high^ subtypes. The GPBAR1^low^ and GPBAR1^high^ patients accounted for 57.21% and 42.78%, respectively. Expression of GPBAR1 in normal liver tissues, liver cirrhosis and HCC was detected with IHC and qPCR (**Figures 1C-1E**), showing that GPBAR1 expression was increased in liver cirrhosis and HCC.

### Correlation between GPBAR1 and other clinicopathological factors

The Chi-square test was applied to investigate the correlation between GPBAR1 and other clinicopathological factors (**Table 1**). The clinicopathological factors included the gender and age of patients, tumor number, tumor size, histopathological grade, vascular invasion, liver cirrhosis, HBV/HCV infection, T stage, N stage, and TNM stage. There are no patients with distant metastasis, so the M stage was excluded. All factors were two-categorized for the Chi-square test. In the analysis, GPBAR1 had no significant correlations with the above factors.

**Table 1:** Correlation between GPBAR1 and clinicopathological factors.

Characters	GPBAR1		*p* ^a^
	Low	High	
Gender			
Female	47	36	0.888
Male	68	50
Age			
<60	90	64	0.524
≥60	25	22
Tumor number			
Single	105	75	0.348
Multiple	10	11
Tumor size (cm)			
≤5	58	38	0.380
>5	57	48
Histopathological grade			
I+II	73	52	0.663
III	42	34
Vascular invasion			
Negative	76	52	0.412
Positive	39	34
Cirrhosis			
Negative	41	25	0.326
Positive	74	61
HBV			
Negative	31	21	0.684
Positive	84	65
HCV			
Negative	107	81	0.745
Positive	8	5
T stage			
I+II	81	59	0.780
III+IV	34	27
N stage			
I	101	72	0.406
II	14	14
TNM stage			
I+II	68	46	0.424
III+IV	47	40

a Chi-square test.

### Prognostic significance of GPBAR1 and other clinicopathological factors

**Figure 2:**
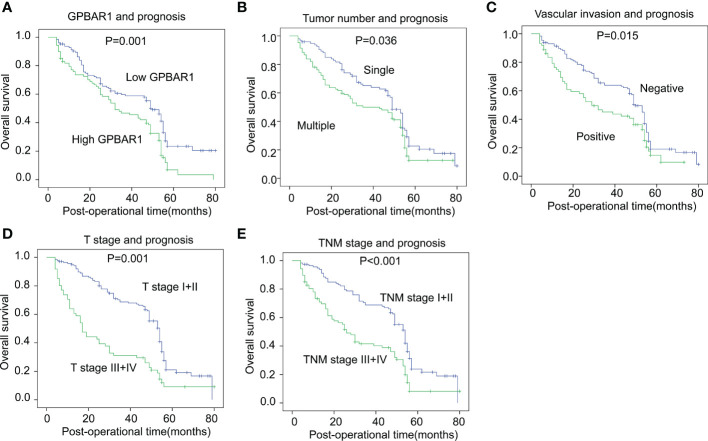
The correlation between GPBAR1 and OS rates. The cohort was divided into subsets according to different parameters. High GPBAR1 **(A)**, multiple tumor numbers **(B)**, positive vascular invasion **(C)**, advanced T **(D)**, and TNM **(E)** stage were all significantly associated with poor prognosis. The statistical significance is analyzed by a log-rank test.

The prognostic significance of GPBAR1 and the enrolled clinicopathological factors were analyzed with survival analysis. The OS rates were analyzed with univariate and multivariate analyses. In the univariate analysis, GPBAR1 was an unfavorable prognostic biomarker of HCC, predicting a poor outcome (**Table 2**). The 5-year OS rates of GPBAR1^low^ and GPBAR1^high^ patients were 23.3% and 6.9%, respectively (**Figure 2A**). In addition to GPBAR1, multiple tumor numbers, positive vascular invasion, advanced T stage, and TNM stage were all significantly associated with a poor prognosis for HCC (**Figures 2B–2E** **)**. Correlations between GPBAR1 and other clinicopathological factors were not observed.

**Table 2:** The prognostic significance of GPBAR1 and other clinicopathological factors.

Characters	5-year survival rate	*p* ^a^	HR	95% CI	*p* ^b^
Gender					
Female	16.2	0.360			
Male	18.1				
Age					
<60	15.9	0.218			
≥60	21.7				
Tumor number					
Single	22.7	0.036	1		
Multiple	12.6		1.05	0.73–1.50	0.790
Tumor size (cm)					
≤5	26.4	0.766			
>5	20.0				
Histopathological grade					
I+II	19.3	0.374			
III	19.9				
Vascular invasion					
Negative	19.0	0.015	1		
Positive	14.6		1.21	0.85–1.73	0.286
Cirrhosis					
Negative	34.7	0.719			
Positive	21.1				
HBV					
Negative	25.2	0.207			
Positive	14.0				
HCV					
Negative	17.2	0.190			
Positive	15.4				
T stage					
I+II	21.8	0.001	1		
III+IV	9.6		2.55	1.76–3.67	<0.001
N stage					
I	19.3	0.571			
II	0				
TNM stage					
I+II	20.8	<0.001			
III+IV	10.6				
GPBAR1					
Negative	23.3	0.001	1		
Positive	6.9		2.08	1.49–2.88	<0.001

a Log-rank test.

b Cox regression model.

The independent prognostic significance of enrolled factors was further validated by multivariate analysis with the Cox regression hazard model. Prognostic parameters in the univariate analysis were further included in the multivariate analysis for independent prognostic significance verification. As a result, high GPBAR1 levels and an advanced T stage were identified as the independent prognostic factors for a poor prognosis. The hazard ratio of GPBAR1 was 2.08, suggesting that the odds of HCC-related death for GPBAR1^high^ patients were 2.08 times those for GPBAR1^low^ patients.

### Correlation between asynchronous metastasis and GPBAR1

Asynchronous metastases of HCC, including intrahepatic metastasis and lung and bone metastases, were particularly focused on during HCC patient follow-up (**Table 3**). Asynchronous liver metastasis during the 5-year follow-up after surgery was 43.78%, while this number in the lung and bone was 19.90% and 12.44%, respectively. Moreover, we analyzed the correlation between GPBAR1 expression and asynchronous metastasis of different involved organs. Interestingly, high GPBAR1 expression in HCC was significantly associated with positive bone metastasis but not with liver or lung metastases. This result suggested that GPBAR1 expression may be involved in bone metastasis but not metastases to other organs.

**Table 3:** Correlation between different metastasis, GPBAR1, and prognosis.

Metastatic organ	Number	Percentage	GPBAR1		*p* ^a^	5-year OS rate	*p* ^b^
			Low	High			
Liver							
Negative	113	56.22%	63	50	0.635	17.7	0.163
Positive	88	43.78%	52	36		16.6	
Lung							
Negative	161	80.10%	92	69	0.967	19.9	0.003
Positive	40	19.90%	23	17		7.7	
Bone							
Negative	176	87.56%	112	64	<0.001	20.6	0.008
Positive	25	12.44%	3	22		0	

a Chi-square test.

b Log-rank test.

**Figure 3:**
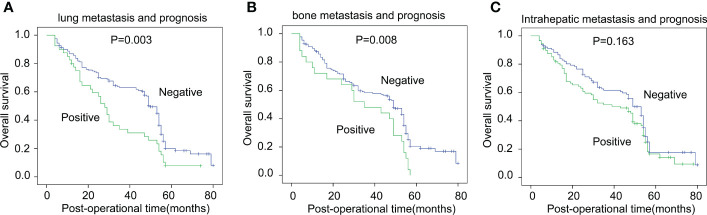
The correlation between asynchronous metastases and OS rates. The correlation between asynchronous metastases of the lung **(A)**, bone **(B)**, liver **(C)**, and OS rates was analyzed with the log-rank test.

In addition, we analyzed the prognostic significance of asynchronous metastasis to the liver, lung, and bone. Lung and bone metastases were significantly associated with poor prognoses in HCC patients (**Figures 3A, B** **)**. The 5-year OS rates of positive and negative lung and bone metastases were 7.7% *vs*. 19.9% and 0% *vs*. 20.6%, respectively. Interestingly, the asynchronous intrahepatic metastasis seemed to have an influence on prognosis, but the effect was statistically insignificant (*p* = 0.163) (**Figure 3C**).

## Discussion

BAs have important physiological functions such as evacuating cholesterol, bilirubin, and hormone derivatives. However, the deregulation of BAs can result in pathological progress such as cholestasis and cancer. The correlation between BAs and HCC has been raised for decades. Current evidence mainly focuses on the fact that BAs in the liver will result in cholestasis, and cholestasis is linked to an increased risk of liver cancer (21). Direct evidence linking BAs or BA receptors and HCC is rare. Moreover, the correlation between BA receptors and HCC is still poorly understood. For a long time, GPBAR1 was mainly considered a regulator of lipid and energy metabolism; here, we demonstrate that GPBAR1 is a prognostic biomarker of HCC for the first time. In previous studies, GPBAR1 expression in the liver was abundant in liver sinusoidal endothelial cells, stellate cells, Kupffer cells, and biliary epithelium cells. Its expression in hepatocytes is the lowest (6). However, its expression and function in HCC have not gained full consensus (7). For the first time, we showed that GPBAR1 expression in HCC is dependent on the time course. GPBAR1 was significantly upregulated in HCC than in liver cirrhosis, with normal liver having the lowest level. In addition, our results suggest that patients with high GPBAR1 are more likely to suffer bone metastasis and a poor outcome. This conclusion indicates that patients with high GPBAR1 expression should get more severe surveillance for tumor recurrence and more intensive precision treatment.

The functions of GPBAR1 in cancer are still very controversial (22). GPBAR1 has been reported to be a tumor suppressor in several cancer types, including gastric cancer and colon cancer (23, 24). However, GPBAR1 promotes progression or correlates with a poor prognosis in some other cancers, including lung cancer, cholangiocarcinoma, and gallbladder cancer (16–18). In HCC, the expression and function of GPBAR1 are poorly studied. Aberrant DNA methylation of GPBAR1 is identified as a potential biomarker for hepatitis B virus-associated hepatocellular carcinoma (25). The expression and molecular function of GPBAR1 in different cancer types may be context- and tissue-dependent. GPBAR1 couples Gαs and activates cAMP-PKA signaling in most cell types, but it is reported to couple both Gαs and Gαi in cholangiocytes (26). More interestingly, functions of GPBAR1 may be dependent on its subcellular localization, as it has been suggested to couple Gαi in the primary cilium of cholangiocytes and Gαs in the apical plasma membrane (26). Moreover, all BAs and many steroids, such as pregnanolone, can activate GPBAR1 (27). GPBAR1 is activated by these ligands and regulates different pathways including PKA, AKT, Src, Rho, mTORC1, NF-κB, and ERK (28). Moreover, GPBAR1 in cholangiocytes, hepatic stellate cells, and macrophages can stimulate different signaling and has various functions (29). These complex GPBAR1 signaling networks confer GPBAR1 more molecular functions in cells, but they also make elucidating GPBAR1 precision function in some special conditions difficult. Since GPBAR1 is involved in many intracellular processes and mediates different signaling pathways, determining how GPBAR1 is activated and what downstream signaling of GPBAR1 is responsible for GPBAR1-associated HCC prognosis remains a difficult task.

Metastasis is a complex, multistep process, and its underlying mechanism is still poorly understood. Tumor metastasis accounts for approximately 90% of all tumor-related deaths (30). HCC has high aggressivity, and most patients suffer from tumor recurrence or metastasis in the course of HCC (31). Intrahepatic metastasis is the most common metastatic site, followed by the lung and other organs. In our study, the prognostic value of metastasis to the liver was not as significant as bone or lung metastasis. Compared with distant metastasis, intrahepatic recurrence has more approaches for treatment, such as lesion ablation or TACE, which may achieve the effect of a radical cure. However, most patients with bone or lung metastasis only receive palliative treatments such as systemic treatments (mainly target therapy or ICI treatment). Different treatment strategies also affect the prognostic significance of metastasis. The metastasis of HCC is a severe threat to patients’ lives, but its molecular mechanism is less studied. Emerging evidence provided new perspectives and brought up the concept of the “pre-metastatic niche,” which remodels the pre-metastatic microenvironment and facilitates tumor metastasis. Obviously, the liver, lung, and bone had different pre-metastatic niches for HCC metastasis. By clinical analysis, we strikingly showed that HCC was more likely to metastasize to the bone instead of the liver or lung. This is an interesting result with great clinical guidance because HCC metastasis to the bone is a rarely studied topic to date. Our results suggest that GPBAR1 overexpression may confer on HCC cells the ability to suit the bone microenvironment but not those of the liver or lung. This is the first report about GPBAR1 and metastasis to a specific organ. This study may provide a new view and help elucidate the molecular mechanism of tumor metastasis.

In summary, we investigated GPBAR1 expression and evaluated its clinical significance by analyzing its association with clinicopathological factors, OS rates, and asynchronous metastases. As a result, we identified GPBAR1 as an independent prognostic factor for HCC and showed that GPBAR1 expression was associated with an increased risk of bone metastasis but not liver or lung metastasis. Our results could provide a new aspect for HCC metastasis studies and help identify high-risk HCC patients, which may ameliorate the prognostic assessment of HCC.

## Data availability statement

The original contributions presented in the study are included in the article/supplementary material. Further inquiries can be directed to the corresponding author.

## Ethics statement

The studies involving human participants were reviewed and approved by Ethics Committee of the Second Affiliated Hospital of Shandong First Medical University and The Affiliated Taian City Central Hospital of Qingdao University. The patients/participants provided their written informed consent to participate in this study.

## Author contributions

NC, JW, LZ, BH and YC collected the specimens and finished the follow-up. NC and JW performed the experiments. NC and ZZ designed the study. ZZ wrote the paper. All authors contributed to the article and approved the submitted version.
